# Implementing an integrated family approach in mental health care for families experiencing complex and multiple problems: a case example in Amsterdam

**DOI:** 10.3389/fpsyt.2024.1409216

**Published:** 2024-08-22

**Authors:** Agnes H. Zegwaard, Frederieke J. Koop, Nico Beuk, Carlinde W. Broeks, Rien L. Van, Carolien Konijn, Aart Franken, Christel M. Middeldorp, Irma M. Hein

**Affiliations:** ^1^ Arkin Youth and Family, Arkin Mental Health Care, Amsterdam, Netherlands; ^2^ Department of Adult Mental Health Care, Arkin Mental Health Care, Amsterdam, Netherlands; ^3^ Department of Youth and Family, Levvel Academic Centre for Child and Adolescent Psychiatry, Amsterdam, Netherlands; ^4^ Departments of Child and Adolescent Psychiatry and Psychosocial Care, Amsterdam UMC, Amsterdam, Netherlands; ^5^ Department of Child and Adolescent Psychiatry, Amsterdam UMC, Amsterdam, Netherlands

**Keywords:** family mental health, family approach, integrated health care, families experiencing complex and multiple problems, cross-domain collaboration, intergenerational transmission of psychopathology

## Abstract

For youth care professionals who work with families with complex needs, we implemented an interagency, family-focused approach involving child and adult mental health care services and child protection services. The primary objective of the collaboration was to minimize fragmentation in service delivery and to improve practitioners’ self-efficacy in supporting families. A total of 50 families were enrolled between 2020 and 2023. Quantitative descriptive analysis was conducted to map the sample characteristics and the correlations between the practitioners’ consultation requests and the recommendations they received. We evaluated the applicability of the model using semi-structured interviews. Results revealed the frequent socioeconomic and psychosocial challenges and co-current mental health issues faced by the families. As expected, practitioners who work with families experiencing complex and multiple problems encountered a range of difficulties in their service delivery. These related to barriers such as poor role demarcation between organizations, practitioners’ unrealistic expectations of other services, the impact of multiple problems on family well-being, and complicated family dynamics. The interprofessional collaboration improved the practitioners’ self-efficacy in supporting families. They also perceived improvements in child safety. The study emphasizes the need for clear pathways for youth care practitioners to obtain assistance from adult mental health services and to liaise with community support and services. It proposes including adults and young people with lived experiences in the interprofessional collaboration. The study data provides initial evidence that the interagency model has added value for youth care professionals who struggle with issues in family-focused care.

## Introduction

Families in contact with child and adult mental health care services and child protection services may be experiencing problems in several areas, including combinations of prolonged socioeconomic and psychosocial challenges and co-current mental health issues. In the Netherlands, such families are defined as families experiencing complex and multiple problems (FECMP) (1). In Amsterdam, child and adult mental health care services and child protection services are mostly separated. Achieving integrated care is difficult, due to lack of coordination, barriers involving separate legal and financial frameworks, differences in perspectives and approach, and siloed clinical practice (2, 3). Barriers to interagency collaboration from the viewpoint of professionals are related to poor role demarcation between organizations, practitioners’ unrealistic expectations of other services, poor communication between organizations, differing perspectives or cultures across professionals and services, difficulties with or a lack of joint budgets, and management and governance issues (3, 4).

Fragmentation of care can result in excessive reliance on health care services by children and parents (5). Research has shown that the burden of mental health issues in children is greater, and tends to persist longer, if their parents are also experiencing mental health problems (6). This relationship is bidirectional, meaning that parental mental health problems and children’s mental health challenges can mutually influence one another (6). Childhood mental health problems have increasingly been linked to adverse social, educational and mental health outcomes later in life (7). Practitioners who work with families encounter problems associated with the impact of multiple problems on family well-being, complicated family dynamics, work with multiple agencies, and high staff turnover. This poses challenges in providing adequate care for these families (4, 8).

Literature points to the need for family-focused practice (FFP), an approach to intervention that emphasizes the family as the focus of attention, as opposed to the individual (9). FFP is defined as intervention provided by health and children’s services to families in which a parent has mental health problems (9). Worldwide there are initiatives aimed at facilitating joint working between adult mental health services and children’s services to improve outcomes, in terms of both service provision and the protection of children and families (9–17). Interdisciplinary and organizational teamwork and interprofessional practice are repeatedly identified as important for achieving a whole-family approach (13, 18).

A recent systematic review identified interprofessional collaboration, with the use of multidisciplinary meetings, as a facilitator to youth care practitioners in adopting a whole-family approach (2). Interagency collaboration that includes such meetings can be a first step toward achieving a coordinated system of care between services, as a stepping stone to more family-focused practice. Multidisciplinary meetings are consultations where professionals share knowledge, highlight concerns and reflect on care processes (2). Research on interagency models has indicated that the insights of experts from different areas, who focus on the current problems in different but interrelated domains within the whole family, can help practitioners understand the multiple problems and the family dynamics (3, 19, 20). Consultation with other professionals also helps to foster better understanding of other services’ strengths and limitations (3, 21). Interprofessional support has been associated with increased self-efficacy of practitioners in supporting families (2, 19).

Interagency collaboration in youth care has been associated with positive client satisfaction, receipt of mental health services, and positive clinical outcomes (3, 5, 18). However, research findings are mixed and, to enable accessible family-focused services in mental health care, it has been recommended to consider *which* components of collaboration actually work for which populations, settings and contexts (2, 12, 18). A recent and unique study developed an initial Program Theory for FFP, which illustrates the interconnectedness between changes that need to co-occur in practitioners, parents and children (22).

As a pilot project, we implemented an interagency family-focused approach in Amsterdam aimed at practitioners working with families in youth care services. The approach engaged multiagency case consultation teams. Reasons for requesting consultation involved difficulties in providing care, which were related to parental mental health problems (including problematic substance use), parenting problems, and concerns about dependent children’s well-being and safety. One of our assumptions was that not every request for help required direct involvement of the adult mental health services. The primary objective of the collaboration was to minimize fragmentation in service delivery and to improve practitioners’ self-efficacy in supporting families. Notably, in the city of Amsterdam there was a perceived need to enhance collaboration between organizations to improve the safety of families after a number of incidents had occurred. Therefore, this case study uses a slightly more risk-focused approach than similar FFP models (14–17). In the limitations section, we describe how the model can be further developed with a strengths-based and capacity building approach, which are recognized as important components in successful delivery of FFP programs (22).

The chief aim of the current study is to develop a better understanding of the use of this multidisciplinary family approach for youth care practitioners working with families. Our community case study focuses on (1) family characteristics in relation to the demand for family-focused care and (2) practitioners’ requests for consultation and the resulting expert recommendations, including engagement of adult mental health services if needed. The study also seeks to contribute valuable information on (3) the experiences of practitioners working in an interagency model as an added value in their work with families. The results can lead to future recommendations and may have implications for clinical practice – enabling interagency collaboration between adult and child services to provide family-focused support for practitioners working with families.

## Method

### Context

Practitioners who requested consultation were experiencing difficulties in service delivery in youth care, which they attributed to an interplay of problems between one or more parents and one or more dependent children. These might involve parents with mental health challenges, instable parenting situations, or concerns about child safety and well-being. Most of the practitioners had shared with the family their need for cross-domain consultation. The families were not directly involved in the multidisciplinary meetings. Multidisciplinary consultation without the involvement of the family and shared decision making do not exclude one another (19). It allows the practitioner to obtain cross-domain recommendations and to comprehend all aspects of the whole family, while still safeguarding the family’s privacy. The family-focused advice enables the professional to better assist clients and parents in making shared decisions. The study procedures were judged by the Ethics Review Board of the Amsterdam UMC and approved. No informed consent from the families or practitioners was needed, because the study design was retrospective, the organizations have implemented an ‘opt-out’ procedure, and the data could not be traced back to the participants.

Excluded were families experiencing serious psychiatric symptoms, such as acute or severe psychoses, acute suicidality, or acute child abuse that required immediate intervention to prevent serious harm to individuals.

#### Setting

With help from municipal grants, we set up a multidisciplinary, interprofessional collaboration in 2019 to enhance multiagency care for families in Amsterdam. Services engaged in the liaisons were facilitating a family approach that integrated adult mental health services (Arkin Mental Health Care), child and adolescent psychiatry (Arkin Youth and Family), integrated youth care and mental health care services (Levvel), child protection services (Jeugdbescherming Regio Amsterdam) and child protection and youth probation services (Partners voor Jeugd, William Schrikker Jeugdbescherming en Jeugdreclassering).

The Arkin Mental Health Care service provides highly specialized mental health care to individuals of all ages in Amsterdam and nearby regions, focusing on a wide spectrum of mental health challenges. Levvel offers comprehensive assistance to children, young people, and biological and foster families in the Amsterdam region. Its services range from parenting support to specialized child and adolescent mental health care, including support for young individuals with mild intellectual disabilities (MID). The regional Child Protection Service becomes involved with a family if there are concerns about child safety; it can take action based on various types of child protection orders. The youth probation service can also act on other court-imposed interventions involving young offenders.

### Key programmatic elements

#### The case consultation teams

The case consultation teams, whose members did not know one another beforehand, were organized top-down. Consistent with research findings about establishing collaboration and the need to familiarize oneself with the services of other professionals, it took several months to create a steady pool of 22 experts, from which a team of ten professionals was drawn for each consultation (2). To ensure that each team would have a balanced representation of experts from Arkin and Levvel with diverse professional backgrounds, the pool was composed of professionals with a broad range of expertise:

1. Adult and child and adolescent psychiatrists and psychotherapists, clinical and other psychologists. These included senior professional supervisors with extensive knowledge of personality disorders, trauma, severe and acute mental health challenges, child development, child emotional disorders, and care avoidance.
2. Systemic therapists, with knowledge of relationship difficulties and complex divorces
3. Behavioral experts, with knowledge of behavioral and emotional issues in children
4. Community psychiatric nurses with considerable experience working with adults with mental health challenges and psychosocial issues. They were employed by Arkin and were working on assignment to child protection and youth probation services.
5. Adults and young people with lived experience who worked at Arkin or Levvel. They improved the quality of care through their insights into clients’ needs and vulnerabilities and into service delivery.
6. Two staff members from the child protection and youth probation agency.

Occasionally, service providers from the domain of social care were invited to participate if they were already involved in a client’s treatment plan. In Amsterdam, the social care domain can provide parental support in upbringing and protection of child safety.

#### Procedures

##### Expert preparation

For each consultation requested by a youth care or mental health practitioner, a team of 4 members of the expert pool – two psychiatrists from Arkin, a clinical child psychologist and psychotherapist from Levvel, and a behavioral expert from Levvel, with secretarial support – managed the planning and commitments of the larger consultation team. The appointed experts provided prior telephone consultation and could assist with the practitioner’s preparation.

##### Practitioner’s preparation

The practitioner’s preparation included completing an online form containing the following information:

- Descriptive information about the perceived family situation and challenges and about the practitioner’s cross-domain consultation request.
- Information from electronic health records of the child or children:

1. Emotional and behavioral problems and mental health care history of the child or children (DSM-5, APA 2013) (23)
2. Emotional and behavioral problems and mental health care history of the parent or parents (DSM-5, APA 2013) (23)
3. Family circumstances, including family composition, well-being of siblings, social support network, finances, housing, ethnic background, work and educational functioning, and family strengths and resilience
4. Children’s adverse childhood experiences, such as complex parental divorce (defined as a divorce with spouses experiencing high conflict), domestic violence or child abuse
5. Estimated child safety, rated on a scale from 1 (“very unsafe”) to 7 (“completely safe”)
6. Any involvement of child protection services
7. Number and types of support and health services involved.

Unique family characteristics were redacted upon receipt of the form, and no names, birth dates or demographic and other identifiable characteristics were shared with the team members.

##### Consultation team preparation

One week before consultation, the team received the redacted form to prepare the meeting.

#### During the meeting

Two unchanging care directors from Arkin and Levvel chaired each meeting. The meeting followed a fixed agenda based on the Balint method online (24): (1) The practitioner began with a brief overview of the family involved, the stagnation, and the request for help. (2) The consultation team asked “what-questions” to clarify the problem. (3) If necessary, the professional reformulated the consultation questions to ensure they were accurately understood and addressed. (4) Hypotheses were formulated by the experts. (5) In line with the hypotheses, advice was formulated, intended to be specific, actionable recommendations at the intervention level. (6) The financial feasibility of a change in the treatment plan was assessed.

#### Practitioner’s actions after the meeting

The family-focused advice enabled the practitioner to better assist the client and the parents in making shared decisions.

**Figure 1:**
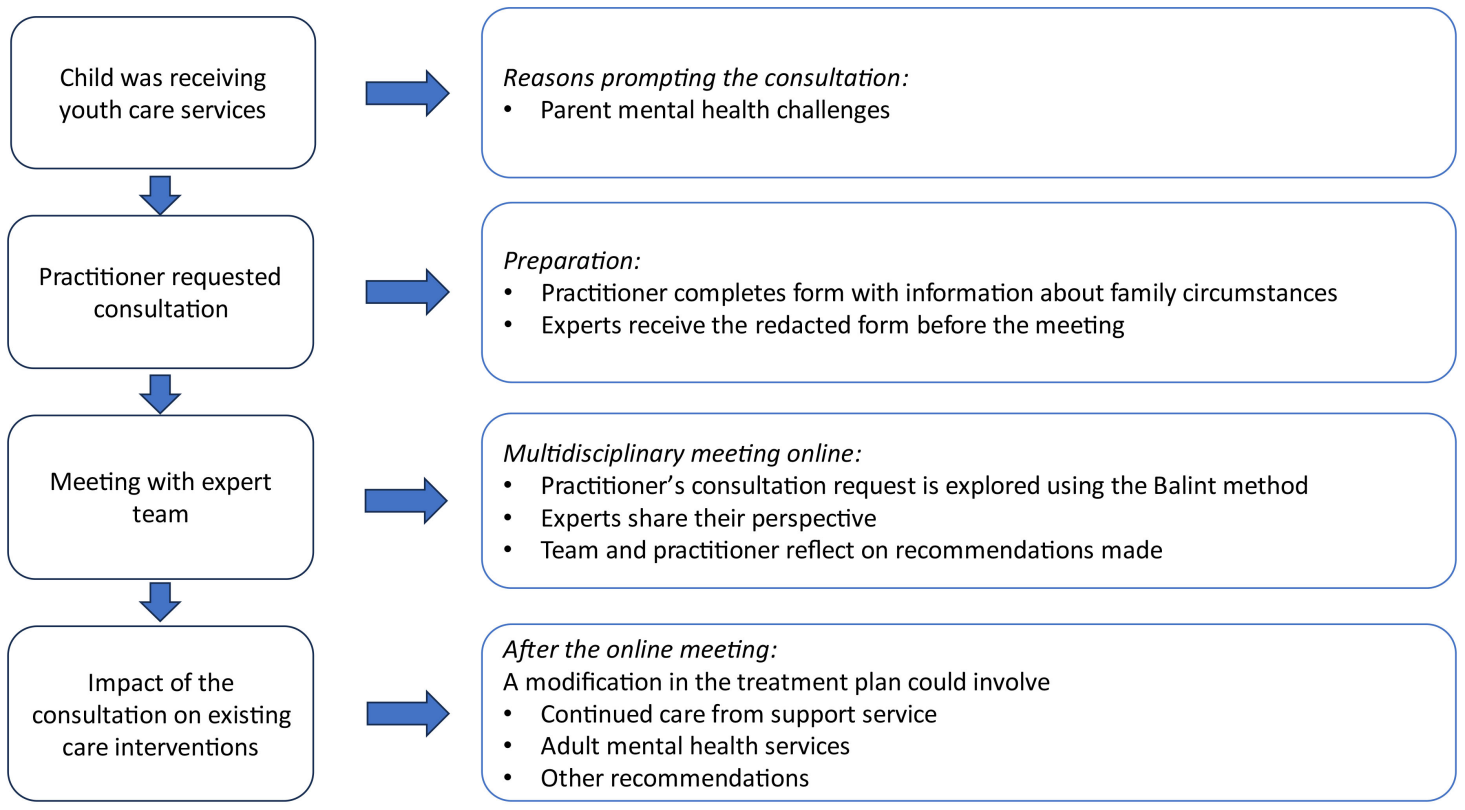
Flowchart of steps in the interagency consultation model.

**Figure 2:**
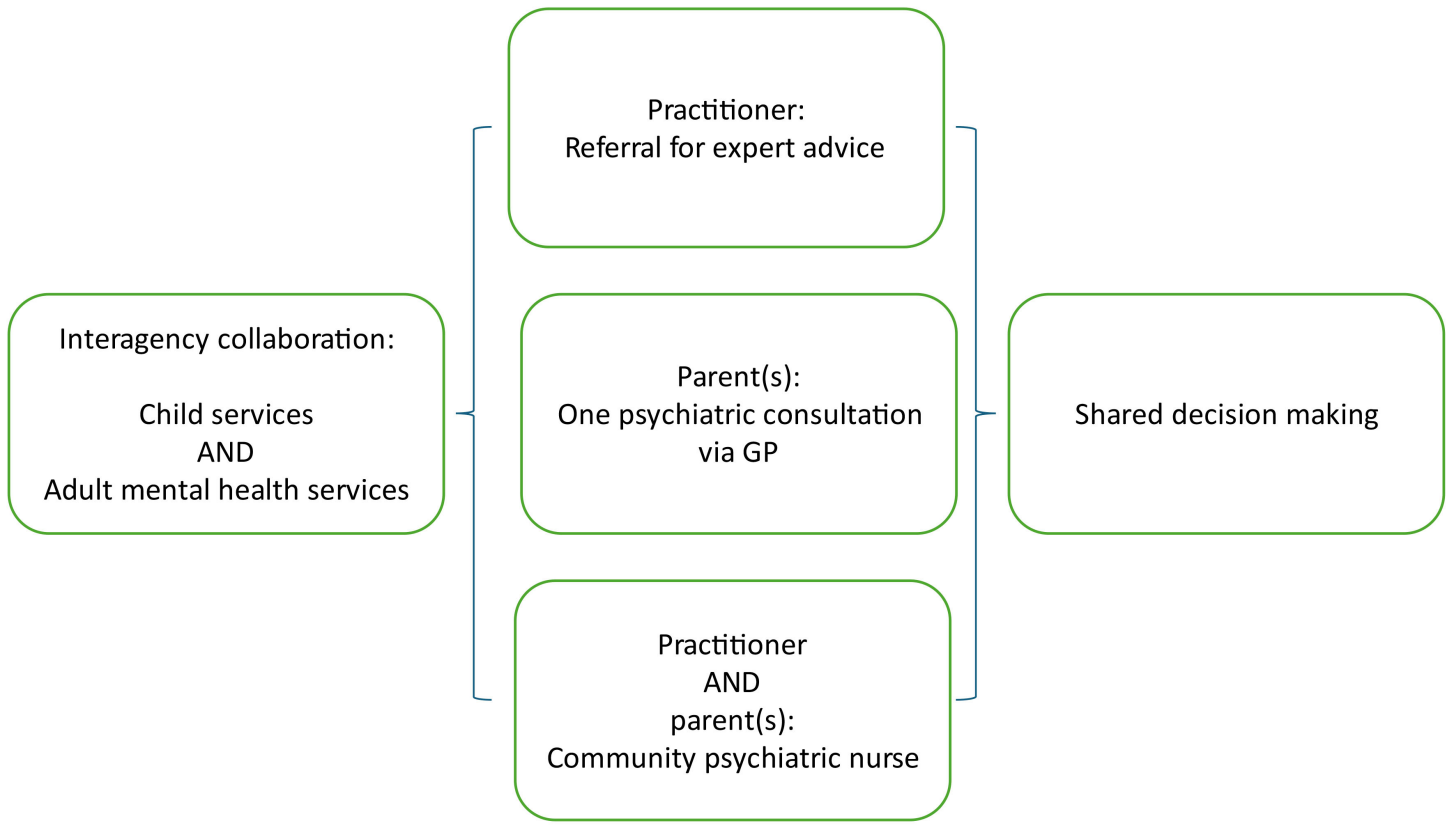
Pathways for youth care practitioners to obtain assistance from adult mental health services, facilitating shared decisions with parents.

**Figure 1** depicts the steps from beginning to end of the procedure for interagency consultation. For practitioners who are advised to obtain assistance from adult mental health services, the pathways to the types of available assistance for practitioners or parents are depicted in **Figure 2**.

#### Data analyses

Between 2020 and 2023, a total of 50 families were discussed in the monthly consultations. Each online meeting lasted 90 minutes: two consecutive consultations of 45 minutes focusing on two practitioners and families. Descriptive data on the families, the discussion and the recommendations were noted on the form by one team member during the meeting. No personal data on families or practitioners was recorded. The form was coded with a number. The code was traceable back to the practitioner, but not to the family. Data from the form was coded with a study ID and entered into SPSS. Data was scored and verified by two independent analysts (C.K, A.F). It was scored using the Classificatie Jeugdproblemen (CAP-J), the Dutch system used to categorize the nature of child and adolescent problems (25). CAP-J serves as a supplement to classification systems focused on disorders, such as DSM-5 (23). Rather than disorders, CAP-J targets issues, and it is specifically tailored to children, adolescents and family and their environmental problems. Quantitative descriptive analyses were then conducted to map the characteristics of the study sample. The practitioners’ consultation requests and the resulting recommendations were categorized thematically by two independent researchers (C.K, A.F), and linear relationships between them were analyzed using Pearson correlation.

Of the first 37 practitioners that requested consultation, 36 were approached for evaluation via an interview at 6 weeks (30 practitioners) and 6 months (14 practitioners) after the consultation. High staff turnover was a reason for sample attrition. The interviews were conducted via video calling, using Microsoft Teams, and lasted about 60 minutes on average. We have utilized semi-structured qualitative interviewing (26). The interview guide was basic, consisting of three main topics: 1) reflection on the consultation model; 2) follow-up; and 3) new actions. A topic list was used for the semi-structured interviews to encourage reflection on the applicability of the consultation (6-point Likert scale from 0 = “not useful” to 5 = “useful”); on perceived change in family functioning based on consultation outcomes (3-point scale from 0 = “not achieved” to 2 = “achieved”); on the degree of goal achievement 6 months later (goal attainment scoring, −1 = “decline” to +2 = “goal achieved”); and estimated child safety (after 6 months; 7-point scale from 1 = “unsafe” to 7 = “safe”).

The answers were noted on an online form. The data from the interviews has been condensed into summaries and broadly categorized based on the predetermined themes of the interview guide and topic list– practitioner’s satisfaction on working with the model, strengths of the model, relevance of the recommendations made, goal achievement, and costs – and on codes that emerged during the analysis – practitioners’ perceived self-efficacy in supporting families, constitution of the expert team, experience working with families, and need for phased and stepped care.

## Results

The results are presented in the order of the research questions: (1) family characteristics, (2) practitioners’ consultation requests and experts’ recommendations, and (3) practitioners’ experiences with the interagency model as an added value in working with families.

### Family characteristics

#### Offspring

**Figure 3:**
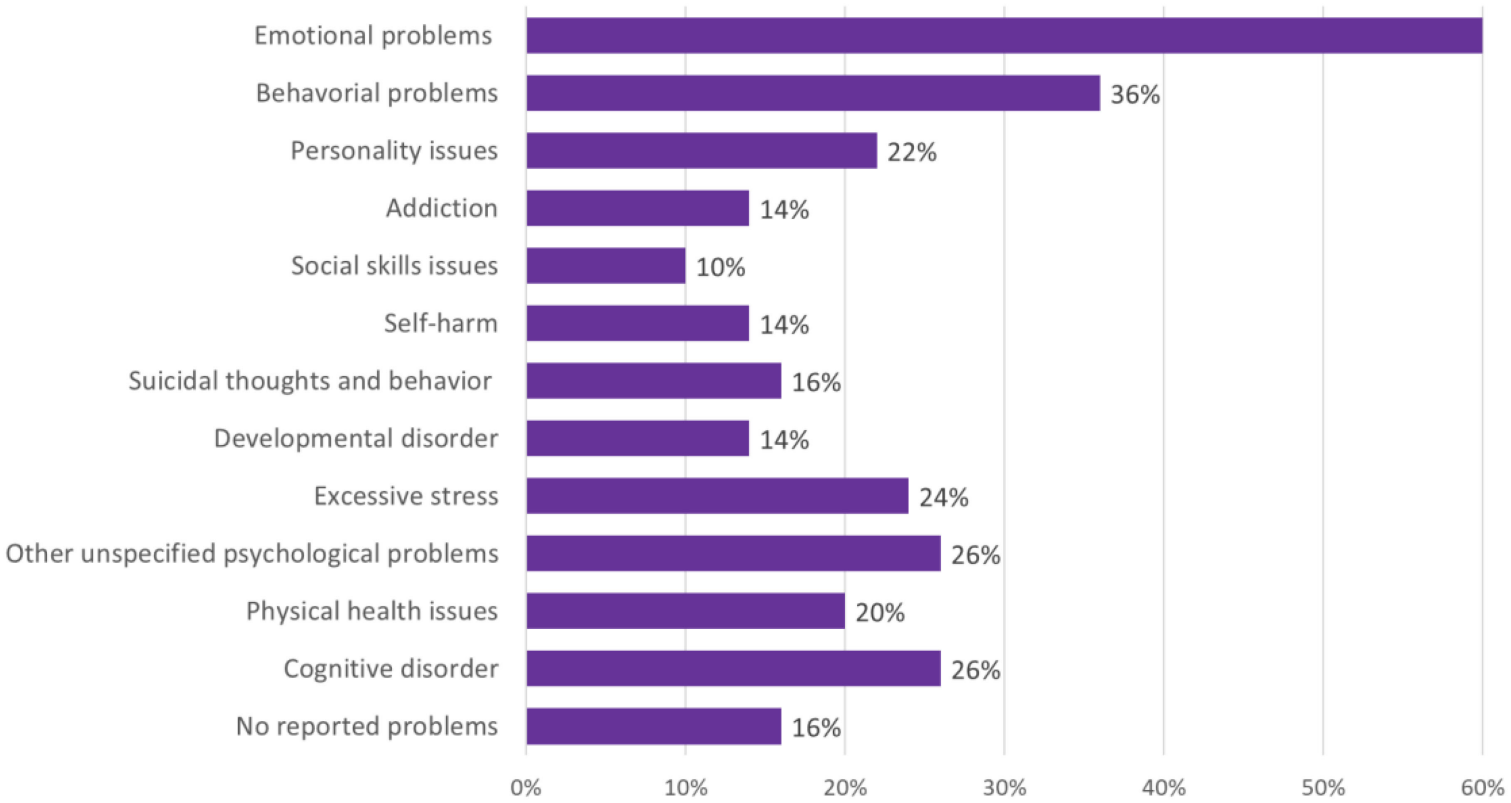
Offspring mental health and behavioral problems, in percentages.

An overview of problems of the families’ offspring is presented in **Figure 3**. A majority of children exhibited emotional and behavioral dysregulation related to mental health challenges. Approximately 62% of the children had received a diagnosis according to the Diagnostic and Statistical Manual of Mental Disorders [DSM; APA 2013 (23)], including conditions such as depressive disorder, unspecified anxiety disorder, unspecified trauma- or stress-related disorder, posttraumatic stress disorder (PTSD), attention deficit hyperactivity disorder (ADHD), borderline personality disorder (BPD) or attachment disorder, oppositional defiant disorder (ODD) and autism spectrum disorder (ASD). Many children were experiencing co-current challenges: anxiety and mood disorders often co-occurred with other disorders, such as trauma-related disorders, PTSD, ASD, ODD or ADHD. Presumably one in four children had a cognitive disorder or mild intellectual disability (MID).

#### Parents

**Figure 4:**
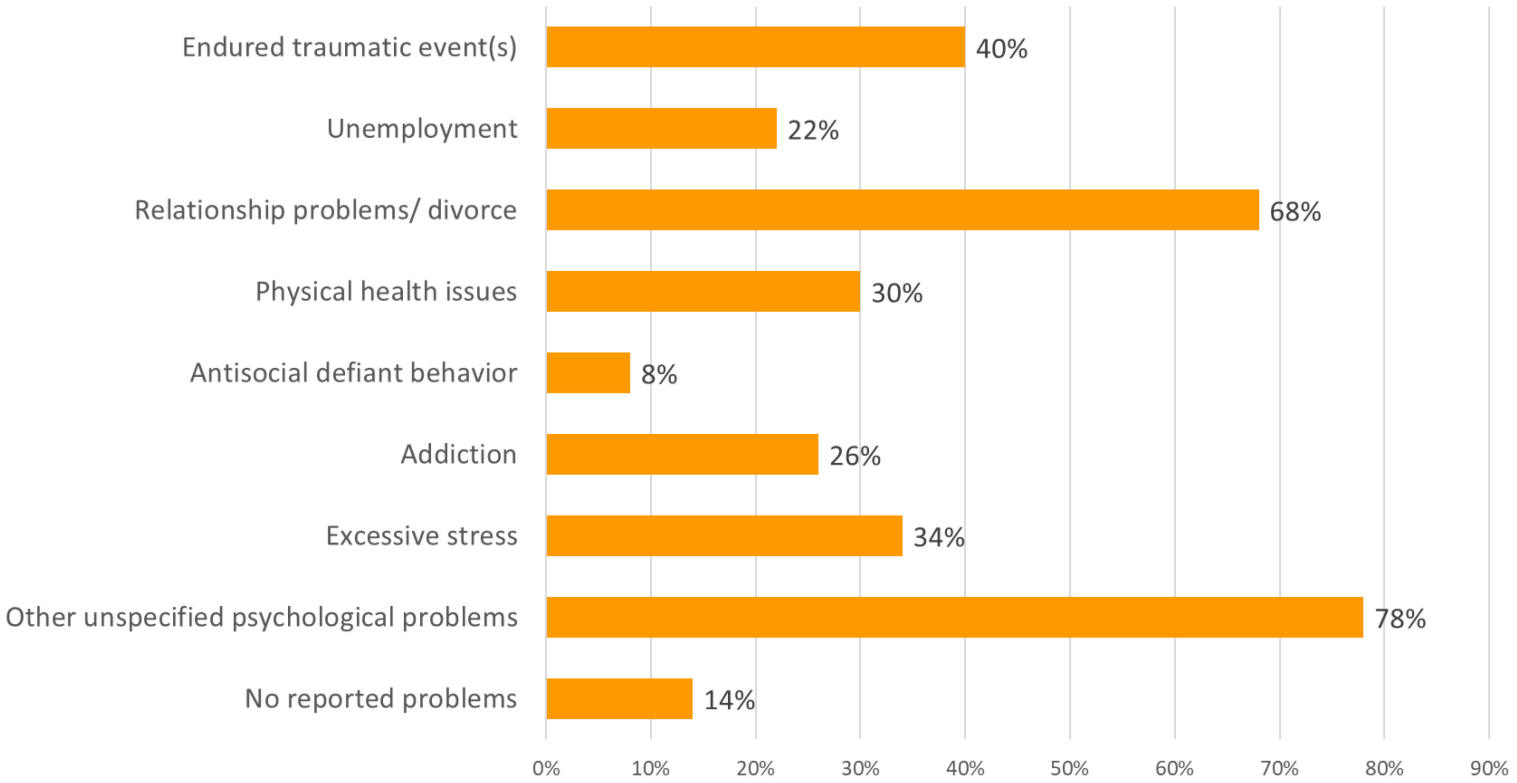
Parents’ problems, in percentages.

An overview of parents’ emotional and behavioral problems is presented in **Figure 4**. Additionally, 48% of the parents had been previously diagnosed with mental disorders as defined by the Diagnostic and Statistical Manual of Mental Disorders (DSM; APA 2013 (23). Parents had experienced conditions such as depressive disorder, anxiety disorder, unspecified trauma- or stress-related disorder, PTSD, ADHD, BPD, psychosis or addiction. Practitioners observed in most parents (78%) current emotional and behavioral dysregulation.

The co-current emotional and behavioral dysregulation of parents and children revealed the interplay of mental health challenges and the psychological overload in families.

#### Family composition and living conditions

The majority of the children of all genders in youth care were in early adolescence, with a mean age of 10.6 and ranging from age 6 to 16. The average number of children per family was 2.7 (compared with an Amsterdam household mean of 1.5 children) (27). Most children lived at home (74%) and some (26%) temporarily in network or foster families. Many children were part of single-parent families (36%) or were living alternately between two parents (16%). Some 34% lived with both parents, compared to 67% in the general population of the Netherlands (28). A significant proportion of the families had migrant backgrounds (32%), compared with 42.3% of non-European origin the Amsterdam general population (29).

**Figure 5:**
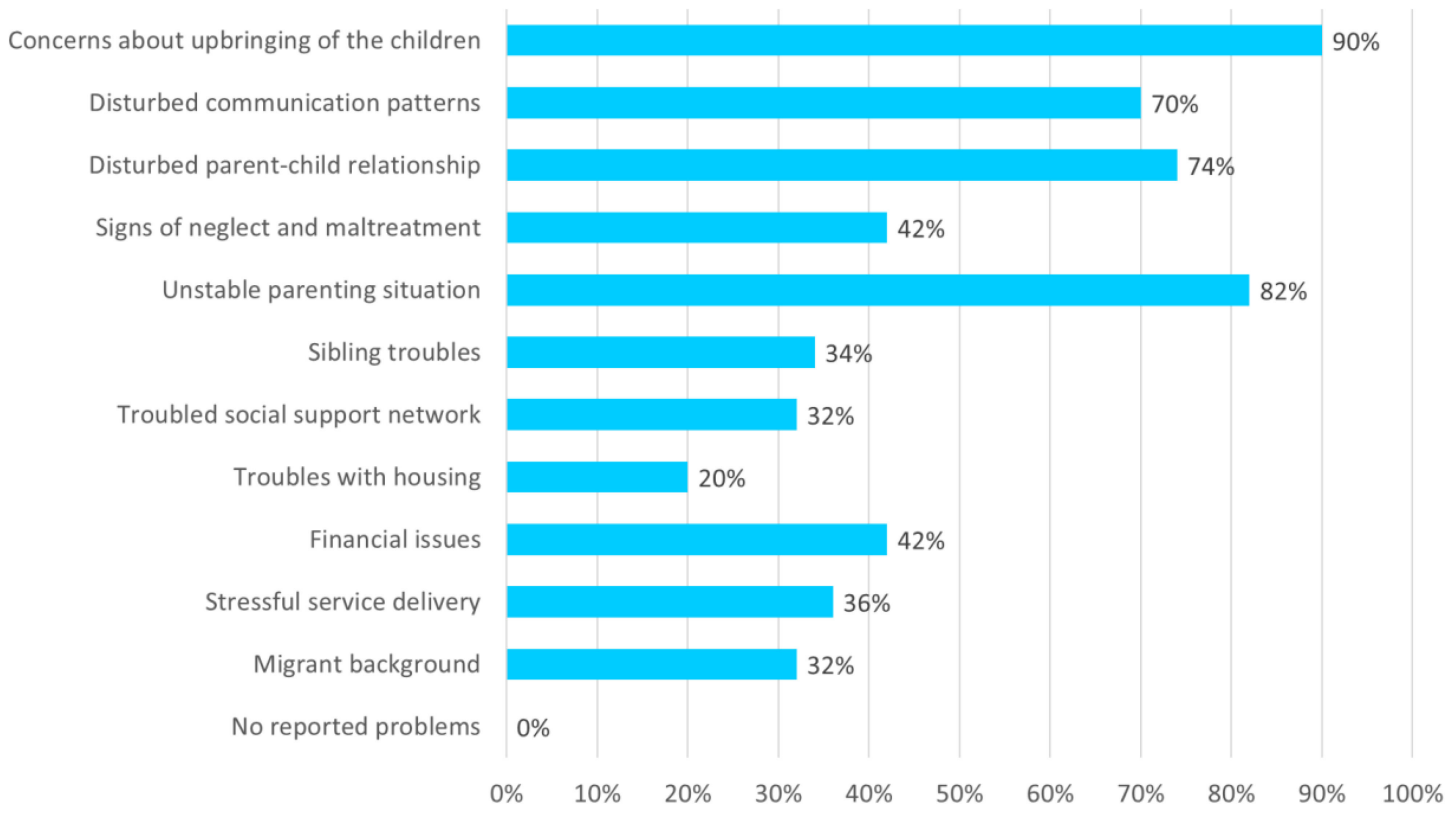
Living conditions and parenting situations.

The data revealed the socioeconomic and psychosocial challenges faced by families, such as social support network issues, low socioeconomic status (SES), and troubles with housing (20%), including risk of eviction, living in too small dwellings, and uncertain housing situations (**Figure 5**). Unemployment and problems at work or school were prevalent, such as absenteeism and dropout and needs for school guidance or special education. If practitioners reported on family resilience, that was related to perceived parental love or to perceived cooperation with service delivery.

#### Parenting situation, child safety and protection services involvement

A partial view of the families’ parenting situations also emerges from **Figure 5**. For nearly every family, there were concerns about the upbringing and overall well-being of the children (90%). Child protection orders were in place in 53% of the families. On a scale from 1 (“low safety”) to 7 (“high safety”), the estimated safety in the families (*N* = 50) averaged 4.6; in 19% of families it was rated between 2 and 3, reflecting significant concerns about the safety and well-being of the children involved. Unstable parenting situations, including relationship and communication difficulties, were common. In 43% of the families, complex divorce problems were reported, for which some families were receiving targeted help (30). The average number of care or support organizations involved per family was 3.7, with a range of 2 to 8. In 36% of the families, the practitioners reported stressful service delivery. Such statistics confirm the complex and challenging circumstances faced by families receiving youth care services, as well as the practitioners’ need for multiagency collaboration.

### Practitioners’ consultation requests and experts’ recommendations

The assumption was that not every request for help would require direct involvement of adult mental health services, despite the complex needs of a family and practitioners’ sometimes mistaken assumptions of a need for adult mental health care.

As expected, practitioners’ consultation requests involved a perceived need for stepped care for parents and/or children (54%), the most appropriate care for the family (31%), improvement of collaboration between organizations (24%), help in securing child safety (17%), support with finances (6%) and practitioners’ self-efficacy (2%).

In line with our assumptions, practitioners were mostly advised to devote more time and energy to engaging the families for care provision (69%). To obtain a better understanding of complex family needs, recommendations were made to gather more information from the family’s general practitioner and from previous health or social care providers (45%) and to involve the family’s social network (45%). Experts also emphasized the need to communicate with cultural sensitivity (29%) and to clarify the families’ needs (16%). For a few families, the experts advised the practitioner to break patient–professional confidentiality (2%) or to consider involuntary care or a child protection order (14%).

Practitioners who received advice to request clearer role demarcation between organizations (43%) were most likely to have requested consultation about improving collaborations between organizations (24%) or about the most appropriate care for the family (31%) (Pearson’s *r* = .40 and *r* = .42, *p* <.05).

Practitioners who enquired about the most appropriate care for children and parents (31%) were also likely to receive recommendations to modify the treatment plan (60%) (Pearson *r* = .41, p <.05): Possible adaptations included the following:

1. A different type of youth care services, or adults or youth with lived experience, might be engaged. Some practitioners were advised to involve public health or social services.
2. For a quarter of the families, the practitioner was advised to seek support from adult mental health services:

- The practitioner could contact a team member from the adult services after the multidisciplinary meeting to discuss whether and in what ways care provision for the parent would be possible.
- The adult mental health service could, after a GP referral, provide a face-to-face consultation with the parent to help in shared decisions about care provision.
- If a family was already in the care of child protection services, the practitioner and the parent could receive a consultation with a community psychiatric nurse, employed by an adult mental health service and working on assignment to a child protection service.

3. Only one recommendation, concerning poor housing conditions, was made to seek support from a different professional domain, even though a large proportion of the families faced socioeconomic challenges like housing, work and financial issues.

### The experiences of practitioners working with the interagency model

At our 6-week follow-up, 88% of the practitioners who had requested a team consultation deemed the recommendations made as helpful and had shared them with the child and parent(s) to facilitate shared decisions. In 65% of the cases, a modification of the treatment plan followed. In some cases, unforeseen developments and/or changes in family dynamics (such as divorce or relocation) had precluded a change in the treatment plan. Practitioners rated the model as applicable for families (3.7 on a scale of 1 to 5) after following the advice given.

At the 6-month follow-up, practitioners rated the perceived child safety as increased (1.6 points higher on the scale of 1 to 7). The practitioner’s satisfaction with the advice given and the modifications in the care provided scored 7.1 on a scale of 10; the goal attainment score was 1.06 (−1 = “decline” to +2 = “goal achieved”).

In summary, practitioners’ reported increased self-efficacy in supporting families and perceived improvements in child safety.

The practitioners judged that the strength of the model for applicability in families lay in (1) the use of heterogeneous experts in a balanced representation, (2) the experts with knowledge of different topics, and (3) the use of a steady expert team. The practitioners valued the model as helpful because (1) the prior preparation, though time-consuming, helped to clarify complexity and the need for cross-domain consultation, and (2) it was possible to address multiple issues simultaneously.

The practitioners judged the strength of the model for cross-domain collaboration by virtue of (1) the perspective of experts from various professional backgrounds, (2) the quantification of child safety, and (3) the clearer role demarcation between the organizations. Those interprofessional perspectives enabled the youth care practitioners to better interpret and cope with parental emotions and behavior without using diagnostic labels. The practitioners also reported an improvement in their own self-efficacy in supporting families.

## Discussion and future recommendations

Our study data have provided the first evidence to our knowledge that the interagency model has added value for professionals working in youth care services as they encounter issues in family-focused care. At 6-week and at 6-month follow-ups, the practitioners reported improvements in their self-efficacy in supporting families experiencing complex and multiple problems (FECMP). Previous research has shown that interprofessional support helped practitioners to maintain a sense of control and a focus on their own expertise and goal achievement (19). As expected, practitioners working with families encountered barriers in service delivery – such as poor role demarcation between organizations, unrealistic expectations of other services, the impact of multiple problems on family well-being, and complicated family dynamics – as well as needs for multi-agency care (3, 4, 8). In families, a “downward spiral” can occur if service delivery does not suit the parental and family needs (4). Practitioners must be aware of the families’ psychological overload due to prolonged socioeconomic and psychosocial challenges, relationship troubles, acculturation problems, learning disabilities, and mental health issues which can co-occur and interact transdiagnostically (8, 9, 20). The perspectives of experts in child and adult mental health, including those with knowledge of complex divorce, helped practitioners to be aware of such dynamics. They could devote time and energy to engaging the families for care provision, and they could be alert to overload arising from the service delivery (4). The interprofessional collaboration between services also made practitioners more aware of the limitations of child and adult mental health services (3). *Active involvement of the family’s GP* may often be lacking in youth care delivery. In the Netherlands, the role of the GP is crucial in providing and coordinating primary care services to patients and referring patients to specialist care when needed.

This study also confirms that sufficient time and resources are needed for interagency collaboration to obtain a better understanding of complex family needs (2, 18). The practitioners’ preparation for the consultation included the help of an expert and the use of a family-focused form to clarify the complexity and the practitioner’s need for cross-domain advice. One facilitator in the consultations is the commitment of a steady, balanced group of heterogeneous experts with a permanent chair (11). A quantification of child safety was reported as helpful when working with services from different domains. The expert perspectives also helped to create realistic expectations about adult mental health service delivery (3). Although this approach requires a substantial time investment, we expect that the benefits will outweigh the costs in the long term. The practitioners’ self-efficacy in supporting families was bolstered, and they also perceived improvements in terms of child safety.

However, it is also important to acknowledge that adult mental health services for one or both parents were indeed deemed necessary in 25% of the families we studied (see **Figure 2**). In interagency collaboration for families, it is important to build clear referral pathways that keep families with complex needs engaged and committed (9, 13). A *referral for expert advice* from an adult mental health service will enhance both the service delivery and the collaboration between child and adult services (3, 9). In Amsterdam, the adult mental health services could, after GP referral, also provide a face-to-face consultation to help practitioners and families make shared decisions about care provision. In Amsterdam, *community psychiatric nurses* can be consulted by youth care practitioners, provided the family is in the care of child protection services. In implementing such additional services into the model, it is important to work with parents, not against them, and to be alert to the tension between “support wanted” and “support provided” (4).

Socioeconomic and psychosocial stressors are a target for intervention in families (9, 20). Such issues might remain unaddressed when practitioners work only with mental health or child protection services. The engagement of *community supports and services* has been suggested as an additional component in interagency collaboration (9).

### Implications for clinical practice

These are some key messages and lessons learnt for implementing a consultation model:

#### For policymakers

- Adequately funded and well-resourced services enable interagency collaboration.
- Engagement of services in a liaison facilitates integrated collaboration.
- Integration of expert referral contacts from an adult mental health service into interagency models and provision of clear pathways for referral to adult mental health services is essential.
- Involvement of community supports and services is recommended.
- The potential of working with adults and young people with lived experience should be considered.

#### For management and senior professionals

- Commitment of a steady, heterogeneous and balanced group of experts is helpful to broaden the perspectives of youth care practitioners.
- Preparation of cross-domain multidisciplinary meetings is aided by use of an information form focused on the whole family.
- Attention should be devoted to role demarcation between organizations and to quantification of child safety.

#### For trainers

- Adequate training should be provided to practitioners to enable understanding of the dynamics of the multiple problems in families.

#### For practitioners

- Time should be devoted to engaging parents for possible mental health service delivery, keeping in mind the tension between “support wanted” and “support provided.”
- Attention should be devoted to socioeconomic and psychosocial challenges and strengths, including the strength of the social support network.
- In cases of complex divorce, a family-oriented systemic intervention can be needed, aimed at reducing parental divorce conflicts.
- An active involvement of the family GP may often be lacking in youth care delivery.

### Acknowledgement of conceptual or methodological constraints

In the model studied here, the families were not directly involved. In-depth analyses were not performed comparing characteristics of the family to the practitioners’ requests for consultation and the expert recommendations received. We therefore cannot assess which types of families might be eligible for consultation with adult mental health services, or what type of practitioners might request such consultation. That would be an interesting research topic for future studies on the model.

Despite our indication that practitioners should identify family strengths, our study remained focused on challenges faced by the families. It lacks any extensive description of resilience. In part this may be explained by an excessively medicalized approach by child and mental health services (4). This suggests that working with adults and young people with lived experience can be needed in interagency collaboration, in order to broaden perspectives to a focus on family strengths and resilience (31).

One review article has indicated that service intervention in families may add to families’ difficulties (4). The present study generated ideas about how the model could be further developed. Because no single issue can be identified as the perpetuating factor in the families’ problems, research is needed on prioritizing what needs to be addressed first for parents, children and family, based on a joint analysis (9). A subsequent study design might include a number of additional focuses: the practitioner’s viewpoint; families’ qualitative priorities or experiences in making shared decisions; quantitative evaluation of the impact of interventions on families’ clinical outcomes and family functioning; families’ knowledge of or involvement in services; and quantification of the impact of interventions on the number of referrals to adult mental health care, social services, and child and youth services; and associations between these factors and financial savings (5, 9).

Unfortunately, specific cost-effect outcomes could not be determined on the basis of the available data from the consultations or follow-ups. We could include no common measures of estimated financial savings, of failures to achieve families’ qualitative priorities, or of their experiences with service delivery (9). Research on the economic evaluation of family-focused programs suggests that a long-term horizon (at least up to early adulthood) needs to be applied in the design of economic evaluations, rather than short-term cost-utility analysis using quality-adjusted life years (32). Future research might additionally focus on practitioners’ self-efficacy in supporting families in relation to their own work context, and on their theoretical background and experience in working with families. This could generate insights into the need for training that could develop practitioners’ abilities to work with families in collaborative partnership (8, 9).

Qualitative research is needed on working with adults and young people with lived experience in interagency collaboration for families. It can assess the potential added value of broadening perspectives and reducing the overly medicalized approach of child and mental health services (33). In the future, early intervention and the proactive use of interagency collaboration is recommended (14, 34).

## Data Availability

The raw data supporting the conclusions of this article will be made available by the authors, without undue reservation.
